# Modulation of behavioral and neurochemical responses of adult zebrafish by fluoxetine, eicosapentaenoic acid and lipopolysaccharide in the prolonged chronic unpredictable stress model

**DOI:** 10.1038/s41598-021-92422-6

**Published:** 2021-07-12

**Authors:** Konstantin A. Demin, Tatiana O. Kolesnikova, David S. Galstyan, Nataliya A. Krotova, Nikita P. Ilyin, Ksenia A. Derzhavina, Nataliia A. Levchenko, Tatyana Strekalova, Murilo S. de Abreu, Elena V. Petersen, Maria Seredinskaya, Yulia V. Cherneyko, Yuriy M. Kositsyn, Dmitry V. Sorokin, Konstantin N. Zabegalov, Mikael S. Mor, Evgeniya V. Efimova, Allan V. Kalueff

**Affiliations:** 1 Institute of Translational Biomedicine SPBU, St. Petersburg State University, St. Petersburg, Russia; 2 Institute of Experimental Medicine, Almazov National Medical Research Centre, Ministry of Healthcare of Russian Federation, St. Petersburg, Russia; 3 Laboratory of Preclinical Bioscreening, Granov Russian Research Center of Radiology and Surgical Technologies, Ministry of Healthcare of Russian Federation, Pesochny, Russia; 4 School of Pharmacy, Southwest University, Chongqing, China; 5 Ural Federal University, Ekaterinburg, Russia; 6 Moscow Institute of Physics and Technology, Moscow, Russia; 7 Neurobiology Program, Sirius University, Sochi, Russia; 8 University of Maastricht, Maasticht, The Netherlands; 9 Granov Russian Research Center of Radiology and Surgical Technologies, Ministry of Healthcare of Russian Federation, Pesochny, Russia

**Keywords:** Neurochemistry, Neuroscience, Emotion, Stress and resilience, Pharmacology

## Abstract

Long-term recurrent stress is a common cause of neuropsychiatric disorders. Animal models are widely used to study the pathogenesis of stress-related psychiatric disorders. The zebrafish (*Danio rerio*) is emerging as a powerful tool to study chronic stress and its mechanisms. Here, we developed a prolonged 11-week chronic unpredictable stress (PCUS) model in zebrafish to more fully mimic chronic stress in human populations. We also examined behavioral and neurochemical alterations in zebrafish, and attempted to modulate these states by 3-week treatment with an antidepressant fluoxetine, a neuroprotective omega-3 polyunsaturated fatty acid eicosapentaenoic acid (EPA), a pro-inflammatory endotoxin lipopolysaccharide (LPS), and their combinations. Overall, PCUS induced severe anxiety and elevated norepinephrine levels, whereas fluoxetine (alone or combined with other agents) corrected most of these behavioral deficits. While EPA and LPS alone had little effects on the zebrafish PCUS-induced anxiety behavior, both fluoxetine (alone or in combination) and EPA restored norepinephrine levels, whereas LPS + EPA increased dopamine levels. As these data support the validity of PCUS as an effective tool to study stress-related pathologies in zebrafish, further research is needed into the ability of various conventional and novel treatments to modulate behavioral and neurochemical biomarkers of chronic stress in this model organism.

## Introduction

Stress potently modulates behavior and physiology, including the neuroendocrine and the immune systems^1,2^ both implicated in psychiatric illnesses, such as anxiety, depression and post-traumatic stress disorder (PTSD)^3,4^. Widespread and severely debilitating, affective disorders represent an urgent unsolved medical problem^5,6^, whose therapy is also complicated due to heterogenic nature, determined by multiple genetic, environmental, and other risk factors^7,8^. Animal models are widely used to study affective pathogenesis, typically involving stress as a common pathogenetic factor^9,10^. One of the most commonly used stress models is chronic unpredictable stress (CUS)^11,12^, which exposes an animal (usually, a rodent) to varying stressors for several weeks^11,13,14^, to evoke ‘affective’ (anxiety- and/or depression-like) states^15^. Behavioral and molecular consequences of CUS typically parallel those observed clinically^16^.

The zebrafish (*Danio rerio*) is a relatively novel model species, rapidly becoming widely used to complement rodent data in stress neurobiology research^17,18^. Zebrafish possess high genetic and physiological homology to humans^19^, especially in terms of their evolutionarily conserved neurotransmitter systems^20,21^ and the central nervous system (CNS) morphology^22,23^. Zebrafish are also used in aquatic CUS protocols adapted from rodent models^24,25^. For example, we have recently established a 5-week CUS protocol in zebrafish, examining behavioral, neurochemical, neuroinflammatory and transcriptomic changes induced by CUS, as well as their potential correction by 1-week antidepressant treatment^26,27^.

**Figure 1:**
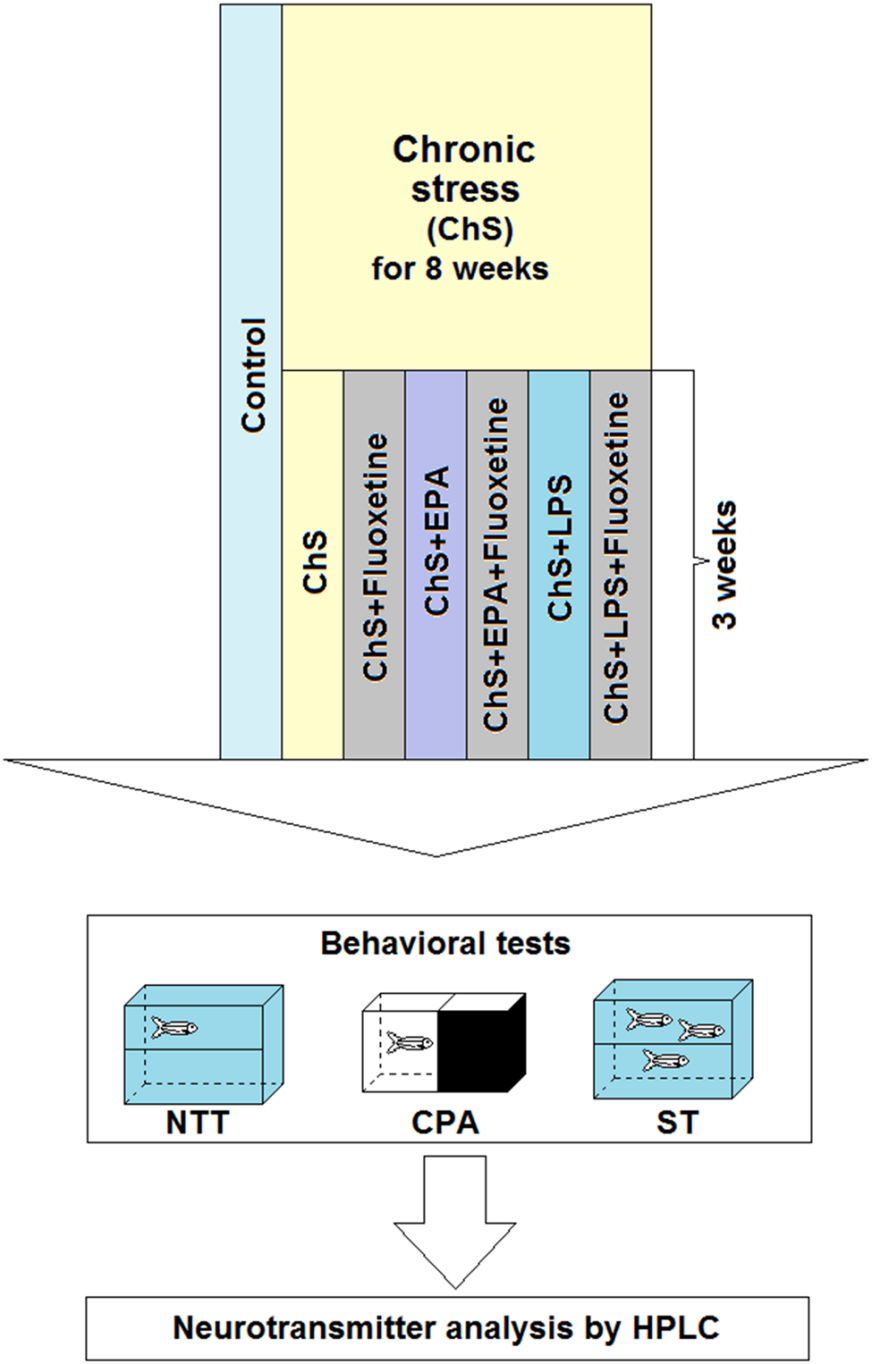
A brief diagram outlining the study experimental design, including the prolonged 11-week chronic unpredictable stress (PCUS) protocol and behavioral testing battery (see Table 1 for details of the PCUS stressors applied in the present study). Abbreviations: *NTT* the novel tank test, *CPA* the conditioned place aversion test, *SH* shoaling test, *EPA* eicosapentaenoic acid, *LPS* lipopolysaccharide, *HPLC* high-performance liquid chromatography.

Importantly, in most clinical cases chronic stress typically lasts longer than 5 weeks, and antidepressant effects take several weeks to occur^28,29^. To address this translational problem, here we develop a novel, prolonged chronic unpredictable stress (PCUS) model, based on rigorous 11-week CUS protocol (Fig. 1 and Table 1) with a 3-week exposure to a conventional antidepressant, a selective serotonin reuptake inhibitor (SSRI) fluoxetine. The effects of this clinically relevant serotonergic antidepressant in the PCUS model were also compared with those of putative positive and negative neuromodulators, such as a neuroprotective omega-3 polyunsaturated fatty acid (PUFA) eicosapentaenoic acid (EPA) and a pro-inflammatory bacteria-derived lipopolysaccharide (LPS), alone or in combinations with fluoxetine.

**Table 1:** Summary of the 11-week prolonged chronic unpredictable stress (PCUS) protocol used in the present study (adapted from^26,27^, with modifications).

Days	Specific CUS stress procedures
1	Three 5-min net chasing sessions with 30-min breaks + predator, two Blue marble gouramis (*Trichogaster trichopterus*) exposure for 24 h in the hometank
2	Three 1-min air exposures and cooling to 10°C for 30 s + exposure to a different zebrafish strain (green GloFish) for 24 h in the hometank
3	Net chasing for 20 min + vortexing for 30 s + darkness for 24 h in the hometank
4	Crowding (10 fish/L) for 6 h + noise (drill sound, 50 db) exposure for 2 h with 30% predator water added into the hometank
5	Crowding/novelty stress in red 8-L bucket (10 fish/L) for 8 h with alarm pheromone + three 1-min electric shocks with 30-min breaks prior to returning to the hometank
6	Three 5-min net chasing sessions with 30-min breaks + food deprivation + alarm pheromone exposure for 24 h in the hometank
7	Vibration (40 Hz) for 2 h + social isolation for 8 h + 30% predator water in the hometank
8	Three 1-min air exposures + shoaling test for 10 min + 30% shallow water with darkness for 24 h in the hometank
9	Three 1-min high temperature (35 °C) exposures with 30-min breaks + 3-cup crowding stress + 60% predator water with 35 novel objects (Kinder surprise toys) for 18 h in the hometank
10	Food deprivation + light–dark box for 8 h + 50% shallow water with bright light (300 lux) for 2 h prior to returning to the hometank
11	Three 1-min electric shocks + three 5-min net chasing sessions with a 3-min break + darkness for 24 h in the hometank
12	Three 1-min cooling + noise for 4 h + predator exposure for 24 h in the hometank
13	Light–dark box for 2 h + three 2-min electric shock sessions + noise for 2 h prior to returning to the hometank
14	Social isolation for 8 h + extra-bright light (1500 lux) for 2 h + 60% predator water added into the hometank
15	Food deprivation for 24 h + three 5-min net chasing sessions with 30-min breaks + 50% predator water exposure in the hometank
16	Three 2-min high temperature exposures + 30 min break + vortexing (10 fish/50-mL tube at 1000 rpm) for 30 s + 0.5 mL/L alarm pheromone exposure for 24 h in the hometank
17	Darkness for 24 h with noise exposure for 6 h in the hometank
18	Shallow water for 8 h (60% of normal water level) + shaking for 30 s + 40% predator water with 35 novel objects in the hometank
19	Three 1-min high temperature exposures, 30 min break + vibration for 4 h + predator exposure for 24 h in the hometank
20	Crowding stress in white bucket for 8 h + three 1-min electric shocks with 30-min breaks prior to returning to the hometank
21	Three 10-min net chasing sessions with 40-min breaks + darkness and food deprivation with an alarm pheromone added for 24 h in the hometank
22	Crowding stress in red bucket with bright-light for 8 h + 60% predator water added in the hometank
23	Three 1-min cooling sessions with 20-min breaks + predator exposure for 24 h in the hometank
24	30% shallow water and bright light for 2 h in the hometank + exposure to 35 novel objects for 24 h in the hometank
25	Shaking in vortex for 30 s with alarm pheromone added + 30-s cooling session + bright light exposure for 6 h in the hometank
26	Noise and vibration for 4 h + novel predator, Oscar fish (*Astronotus ocellatus*) exposure for 8 h prior to returning to the hometank
27	3-cup crowding (12 fish/0.5-L cup) for 6 h under bright light + shallow water (50% of the original, normal level) for 16 h in the hometank
28	Social isolation for 8 h + net chasing for 15 min + 30% predator water added into the hometank
29	Three 1-min air exposures with 30-min breaks + *Astronotus ocellatus* for 2.5 h + *Trichogaster trichopterus* for 2.5 g + alarm pheromone 7 times for 15 min each, prior to returning to the hometank
30	Shaking in vortex for 30 s + darkness with food deprivation for 24 h in the hometank
31	Crowding in red bucket for 8 h + exposure to a different zebrafish strain (green GloFish) for 14 h prior to returning to the hometank
32	Net chasing for 15 min + local hypothermia (22 °C) 2 h + shallow water (40%) exposure for 24 h in the hometank
33	3-cup stress for 6 h + 35 novel objects for 24 h in the hometank
34	Light–dark box for 5 min + three 1-min air exposures with 10-min breaks + predator *Trichogaster trichopterus* exposure for 24 h in the hometank
35	Shaking in vortex for 30 s + shallow water (60%) for 18 h with alarm pheromone added 5 times, with 15-min intervals, prior to returning to the hometank
36	Three 1-min electric shocks + vibration for 4 h + predator *Trichogaster trichopterus* exposure for 24 h in the hometank
37	Food deprivation for 24 h + 50% predator water + alarm pheromone 5 times with 20-min intervals prior to returning to the hometank
38	Shaking in vortex for 30 s + three 1-min high temperature exposures with 30-min breaks + three 5-min net chasing sessions with 30-min breaks + extra-bright light exposure for 20 min in the hometank
39	Three 1-min electric shocks + three 30-s cooling sessions with 30-min breaks + darkness for 24 h in the hometank
40	Social isolation for 8 h + food deprivation for 24 h + vibration for 2 h, prior to returning to the hometank
41	Crowding in red bucket for 6 h + shoaling test for 10 min + extra-bright light for 20 min in the hometank
42	Three 1-min air exposures with 10-min breaks + *Astronotus ocellatus* for 2.5 h + *Trichogaster trichopterus* for 2.5 h, prior to returning to the hometank
43	Net chasing for 20 min + extra-bright light for 2 h + shallow water (40%) exposure for 24 h, prior to returning to the hometank
44	Noise exposure for 4 h + mild hypothermia (22 °C) for 2 h and predator (*Trichogaster trichopterus*) for 24 h in the hometank
45	3-cup crowding for 6 h + 60% predator water with novel objects for 18 h in the hometank
46	Net chasing for 20 min + cooling for 30 s + darkness with food deprivation for 24 h in the hometank
47	Crowing in white bucket for 8 h + vibration and noise for 6 h, prior to returning to the hometank
48	Three 1-min electric shocks with 30-min breaks + exposure to a different zebrafish strain (green GloFish) for 24 h in the hometank
49	Shoaling test for 5 min + social isolation for 15 min + noise for 4 h + net chasing for 20 min, prior to returning to the hometank
50	Light–dark box for 5 min + 3-cup crowding for 6 h + alarm pheromone for 24 h in the hometank
51	Three 1-min air exposure with 1-min electric shock 10 min break + 60% predator water with novel objects for 24 h in the hometank
52	Cooling for 30-s + shaking in vortex with alarm pheromone for 30 s + bright light for 6 h in the hometank
23	Noise exposure for 2 h + vibration for 2 h + social isolation for 4 h, prior to returning to the hometank
54	Three 5-min net chasing sessions with 30-min breaks + extra-bright light for 30 min + exposure to *Astronotus ocellatus* for 2.5 h + *Trichogaster trichopterus* for 2.5 h, prior to returning to the hometank
55	Shaking in vortex for 40 s + three 2-min high temperature exposures with 30-min breaks + darkness for 24 h in the hometank
56	Shallow water (30%) under bright light for 2 h + shoaling test for 10 min + food deprivation for 24 h in the hometank
57	Light–dark box for 12 h + three 2-min electric shock sessions with 30-min breaks + 15-min net chasing, prior to returning to the hometank
58	Two-min cooling sessions with a 20-min break + predator (*Trichogaster trichopterus*) exposure for 24 h in the hometank
59	Shaking in vortex for 30 s + vibration and noise for 6 h + darkness for 18 h in the hometank
60	3-cup crowding for 6 h + 60% predator water with an alarm pheromone and novel objects for 18 h in the hometank
61	20 min net chasing + 2.5 h exposure to *Trichogaster trichopterus* + 2.5 h to *Astronotus ocellatus* + exposure to a different zebrafish strain (*green* GloFish) for 24 h in the hometank
62	Food deprivation for 24 h + social isolation for 8 h + bright light for 2 h, prior to returning to the hometank
63	Three 1-min electric shock exposures with 30-min breaks + light–dark box for 8 h, prior to returning to the hometank
64	Three 1-min air exposures with 30-min breaks + 50% shallow water for 12 h + alarm pheromone added into the hometank
65	Noise and vibration for 6 h + net chasing for 20 min, prior to returning to the hometank
66	Net chasing for 20 min, a 30-min break + vortexing 30 s + darkness and food deprivation for 24 h with an alarm pheromone in the hometank
67	Bright light exposure for 2 h + * green* GloFish exposure with novel objects for 24 h in the hometank
68	3-cup crowding for 6 h + 3 1-min high temperature (35 C) + 30% predator water for 18 h
69	Shaking in vortex with alarm pheromone for 40 s + noise for 4 h + three 30-s cooling sessions with 10-min breaks, prior to returning to the hometank
70	Noise and vibration for 4 h + *Trichogaster trichopterus* exposure for 18 h in the hometank
71	Three 1-min electric shocks with 30-min breaks + two 1-min air exposures + shallow water (30%) exposure for 6 h in the hometank
72	Crowding in red bucket for 6 h + shoaling test for 10 min + alarm pheromone exposure for 18 h in the hometank
73	Shallow water (60%) with bright light for 8 h + three 1-min air exposures with 30-min breaks + vibration for 2 h, prior to returning to the hometank
74	*Astronotus ocellatus* exposure for 3 h + *Trichogaster trichopterus* exposure for 3 h + net chasing for 20 min, prior to returning to the hometank
75	3-cup crowding for 6 h + noise exposure for 2 h + 60% predator water with novel objects in the hometank
76	Vibration for 4 h + darkness with an alarm pheromone exposure for 24 h in the hometank
77	Food deprivation for 24 h + social isolation for 6 h + three 1-min electric shocks with 30-min breaks + vortexing for 30 s, prior to returning to the hometank
78	Behavioral testing in the novel tank test prior to returning to the hometank
79	Training zebrafish in the conditioned place aversion test prior to returning to the hometank
80	Behavioral testing in the conditioned place aversion test prior to returning to the hometank
81	Behavioral testing in the shoaling test, prior to sacrificing the fish and collecting brain samples one day later

## Results

### Behavioral studies

**Figure 2:**
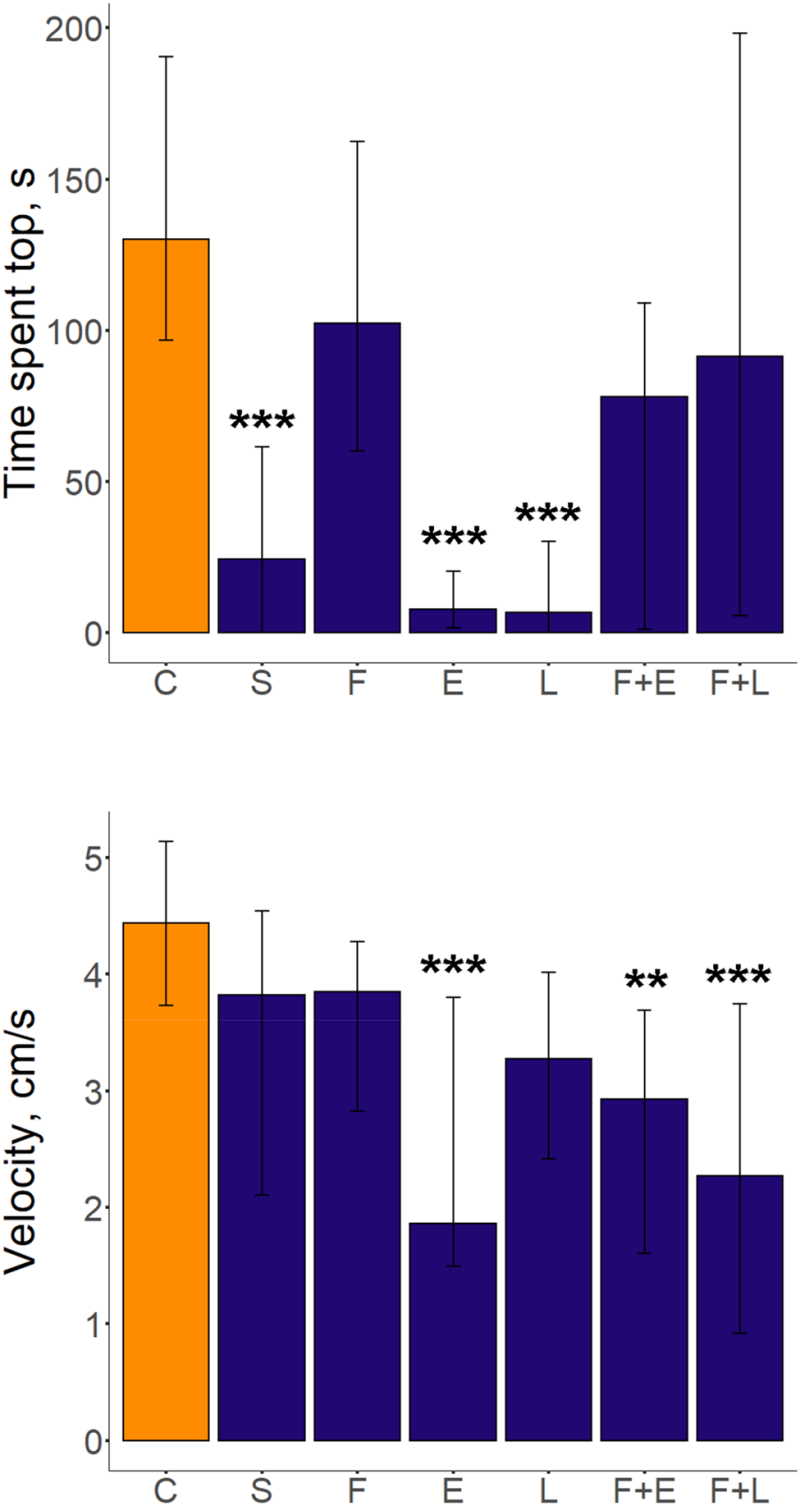
Behavioral alterations induced by prolonged chronic unpredictable stress (PCUS) exposure and fluoxetine, EPA, or LPS treatment in adult zebrafish tested in the novel tank test. Data are presented as median and Q1, Q3 (n = 12–22 per group). *p < 0.05, **p < 0.01, ***p < 0.001 vs. control, post-hoc Dunn’s test for significant Kruskal–Wallis data. Graphs were constructed using the ggplot2 R package^30^ (also see Table 2 and Supplementary Table S1 for statistical details). Groups: *C* control, *S* PCUS, *F* fluoxetine, *E* eicosapentaenoic acid (EPA), *L* lipopolysaccharide (LPS).

In the novel tank test (NTT), the PCUS protocol produced several significant treatment effects, summarized in detail in Table 2 and Supplementary Table S1. Overall, the EPA, fluoxetine + EPA and fluoxetine + LPS exposure significantly reduced zebrafish swim velocity (*p* < 0.001 vs. control, Dunn’s test, Fig. 2). Stress, EPA and the LPS exposure reduced time in top of the tank vs. control (*p* < 0.001 for all groups, Dunn’s test) and vs. fluoxetine-treated groups (*p* < 0.01 for all groups, Fig. 2, Table 2 and Supplementary Table S1). Stress exposure also increased the latency to enter the top vs. both control (*p* < 0.05, Dunn’s test) and fluoxetine groups (*p* < 0.01), whereas LPS increased it only compared to fluoxetine-treated group (*p* < 0.05, Fig. 2, Table 2 and Supplementary Table S1). Finally, fluoxetine + LPS exposure reduced the number of top entries (*p* < 0.05 vs. control group, Dunn’s test, Fig. 2, Table 2 and Supplementary Table S1).

**Table 2:** Summary of the Kruskal–Wallis test results for behavioral and neurochemical alterations induced by the prolonged chronic unpredictable stress (PCUS) exposure and fluoxetine, EPA or LPS treatments in adult zebrafish brain (see also Figs. 2, 3 and 4 for graphical representation and Supplementary Tables S1–S2 for post-hoc test results). Abbreviations: *DOPAC* 3,4-dihydroxyphenylacetic acid, *5-HIAA* 5-hydroxyindoleacetic acid, *NS* not significant (*p* > 0.05).

Parameters	H	df	*p* value
**The novel tank test**			
Velocity, cm/s	30.84	6	*p* < 0.0001
Time spent in top, s	41.81	6	*p* < 0.0001
Latency to top, s	32.65	6	*p* < 0.0001
Number of top entries	22.11	6	*p* < 0.01
**The shoaling test**			
Average inter-fish distance, cm	162.25	6	*p* < 0.001
**Conditioned place aversion**			
Time spent in light, s	36.86	7	*p* < 0.0001
**Neurochemical analyses**			
Norepinephrine, pg/mg	20.45	6	*p* < 0.005
Dopamine, pg/mg	18.70	6	*p* < 0.005
DOPAC, pg/mg	7.49	6	NS
Serotonin, pg/mg	34.71	6	*p* < 0.0001
5-HIAA, pg/mg	33.11	6	*p* < 0.0001
**Monoamine metabolism ratios**			
5-HIAA to serotonin ratio	33.31	6	*p* < 0.0001
DOPAC to dopamine ratio	24.76	6	*p* < 0.0005

**Figure 3:**
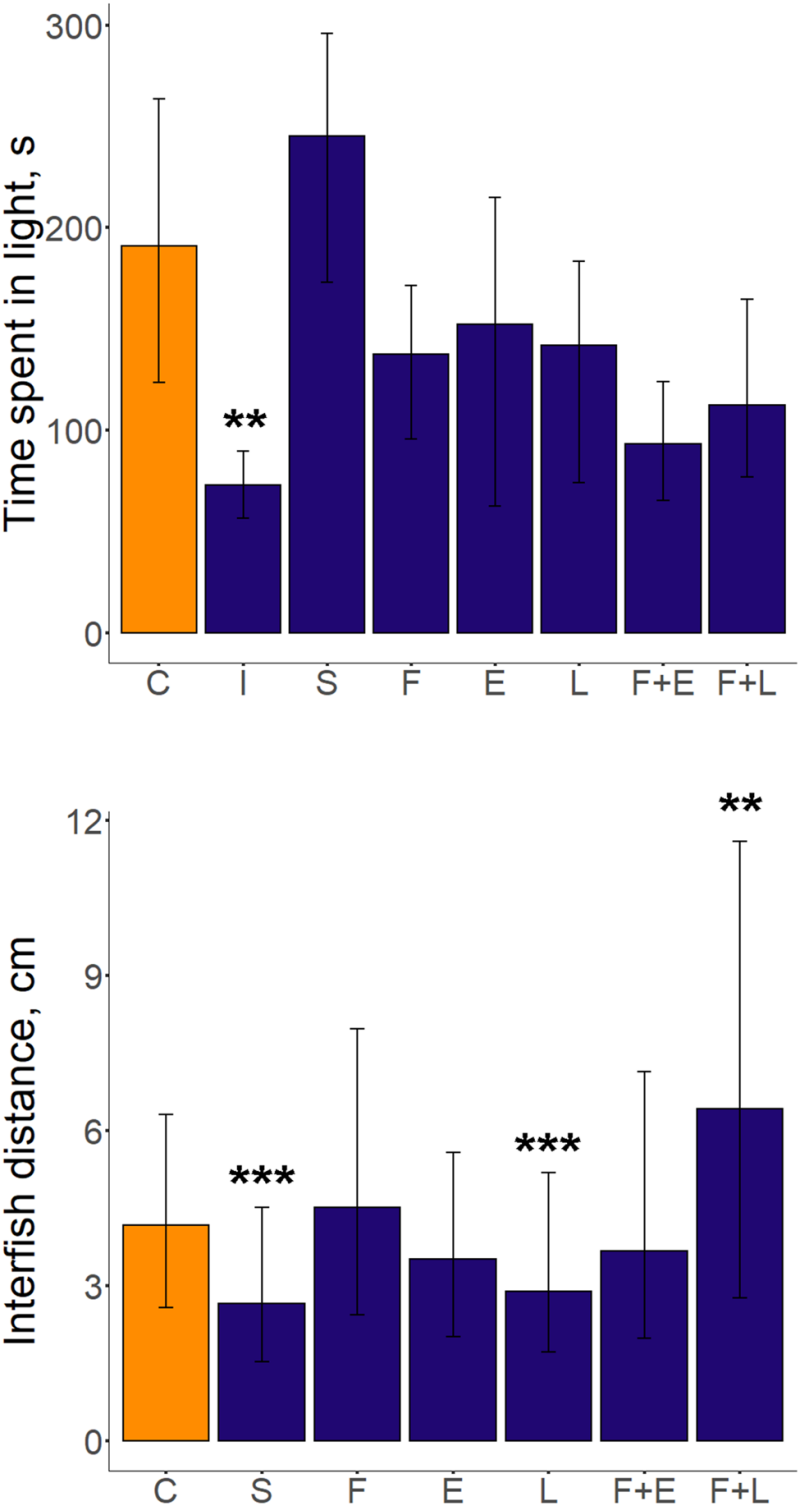
Behavioral alterations induced by prolonged chronic unpredictable stress (PCUS) exposure and fluoxetine, EPA, or LPS treatment in adult zebrafish tested in the shoaling test (ST; inter-fish distance) and conditioned place avoidance (CPA, time spent in the light compartment). Data are presented as median and Q1, Q3 (total number of still images: 198–541 per group for ST (calculated as distances between each zebrafish in each captured still image, thus representing the number of images taken), n = 12–22 per group for CPA). *p < 0.05, **p < 0.01, ***p < 0.001 vs. control, post-hoc Dunn’s test for significant Kruskal–Wallis data. Graphs were constructed using the ggplot2 R package^30^, also see Table 2 and Supplementary Table S1 for statistical details). Groups: *C* control, *S* PCUS, *I* intact (no learning), *F* fluoxetine, *E* eicosapentaenoic acid (EPA), *L* lipopolysaccharide (LPS).

Conditioned place aversion (CPA) protocol was efficient in inducing avoidance learning in the zebrafish, as assessed by time spent in light in intact (non-learning) fish (*p* < 0.01 vs. control group, Dunn’s test, Fig. 3, Table 2 and Supplementary Table S1). While no behavioral alterations were observed in experimental vs. control groups (*p* > 0.05), the PCUS group spent more time in light than intact fish (*p* < 0.0001, Dunn’s test), as well as fluoxetine-treated (*p* < 0.05), fluoxetine + EPA (*p* < 0.001) and fluoxetine + LPS groups (*p* < 0.05, Fig. 3, Table 2 and Supplementary Table S1).

In the shoaling test (ST), PCUS and LPS exposure both reduced average inter-fish distance (*p* < 0.0001 vs. control group, Dunn’s test, Fig. 3, Table 2 and Supplementary Table S1). Furthermore, PCUS exposure decreased inter-fish distance vs. all other groups, except for LPS (*p* < 0.01, Dunn’s test, Fig. 3, Table 2 and Supplementary Table S1). In contrast, fluoxetine + LPS exposure significantly increased the average inter-fish distance in this test, compared to every other group (*p* < 0.05), fluoxetine reduced the shoal cohesion vs. EPA and LPS (*p* < 0.0001), whereas fluoxetine + EPA increased the inter-fish distance compared to LPS (*p* < 0.01, Dunn’s test, Fig. 3, Table 2 and Supplementary Table S1). No other behavioral effects were observed for all endpoints in the NTT, CPA and ST tests between different experimental groups used in this study (*p* > 0.05, NS, Fig. 3, Table 2, Supplementary Table S1).

### Neurochemical analyses

**Figure 4:**
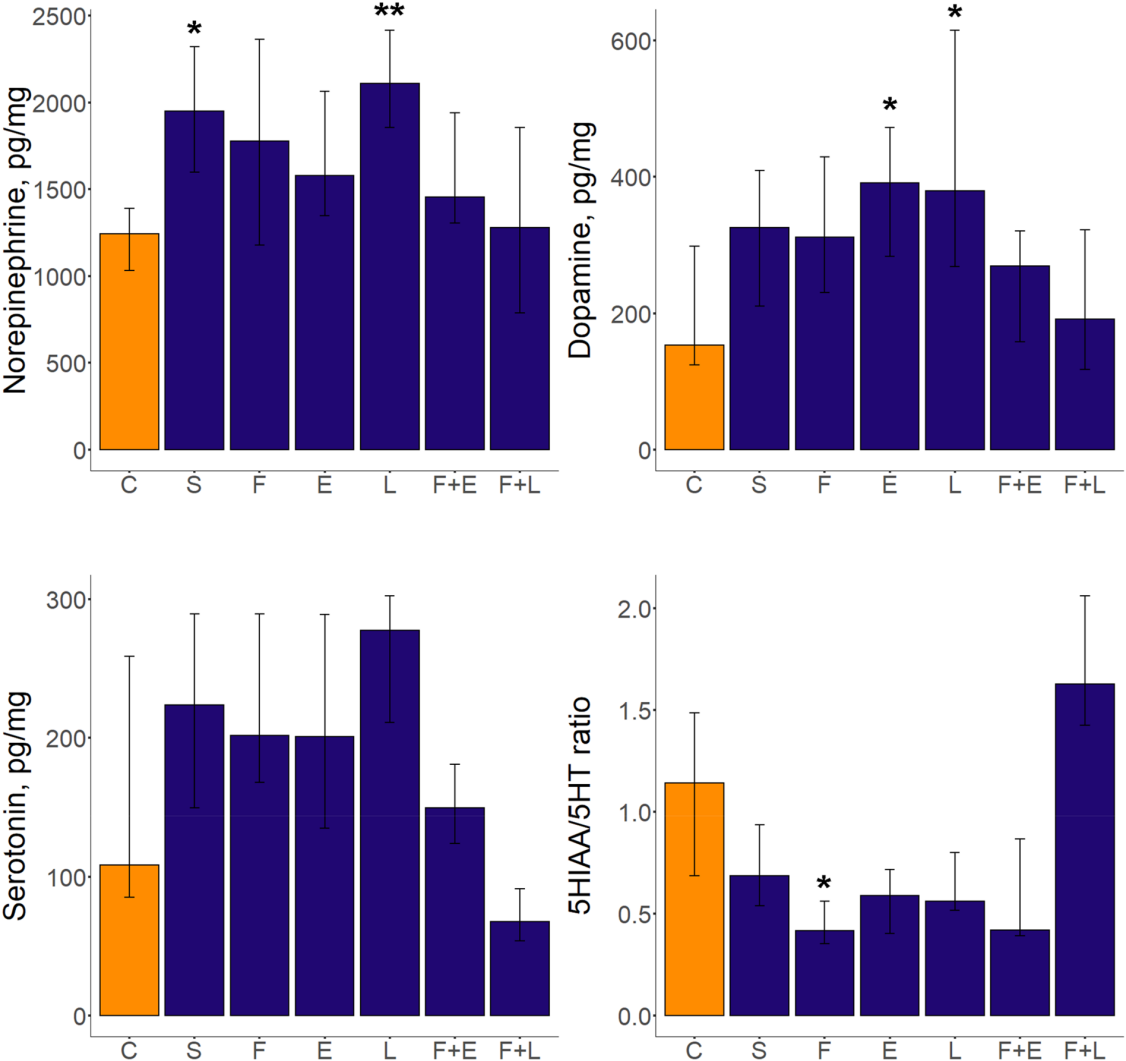
Neurochemical alterations induced by the prolonged chronic unpredictable stress (PCUS) exposure and fluoxetine, EPA, or LPS treatment in adult zebrafish brain assessed using HPLC. Data are presented as median and Q1, Q3 (n = 12 per group). *p < 0.05, **p < 0.01 vs. control, post-hoc Dunn’s test for significant Kruskal–Wallis data. Graphs were constructed using the ggplot2 R package^30^, also see Table 2 and Supplementary Table S2 for statistical details. Groups: *C* control, *S* PCUS, *F* fluoxetine, *E* eicosapentaenoic acid (EPA), *L* lipopolysaccharide (LPS).

Significant treatment effects on zebrafish neurochemical parameters are presented in Table 2 and Supplementary Table S2. Overall, PCUS and LPS both increased levels of norepinephrine in zebrafish brain (*p* < 0.05 and *p* < 0.01 vs. control group, respectively, Dunn’s test, Fig. 4, Table 2 and Supplementary Table S2). Chronic fluoxetine also lowered the 5-hydroxyindoleacetic acid (5HIAA)/serotonin ratio (*p* < 0.01 vs. control) that reflects serotonin turnover, whereas fluoxetine + EPA increased the 5HIAA levels (*p* < 0.05 vs. control), without altering serotonin turnover. Moreover, both LPS and EPA increased dopamine levels (*p* < 0.05 vs. control group, Dunn’s test, Fig. 4, Table 2 and Supplementary Table S2).

Likewise, we found pronounced neurochemical differences between the experimental groups, since both fluoxetine and fluoxetine + EPA groups show reduced 5HIAA levels compared to the stress fish (*p* < 0.05 and *p* < 0.0001, respectively), whereas fluoxetine + EPA fish display lowered 5HIAA vs. the LPS group (*p* < 0.001, Dunn’s test, Fig. 4, Table 2 and Supplementary Table S2). Interestingly, fluoxetine + LPS markedly reduced serotonin levels in zebrafish brain vs. PCUS, fluoxetine alone, fluoxetine + LPS (*p* < 0.01) and LPS groups (*p* < 0.001, Dunn’s test, Fig. 4, Table 2 and Supplementary Table S2). Furthermore, fluoxetine + LPS also elevated serotonin turnover vs. fluoxetine, EPA, LPS and fluoxetine + EPA groups (*p* < 0.0001, *p* < 0.01, *p* < 0.05 and *p* < 0.001, respectively, Dunn’s test). Finally, fluoxetine + LPS increased dopamine turnover (assessed here as the 3,4-dihydroxyphenylacetic acid (DOPAC)/dopamine ratio) vs. EPA and LPS groups (*p* < 0.01, Dunn’s test, Fig. 4, Table 2 and Supplementary Table S2), with no other differences observed between the groups (*p* > 0.05, NS).

## Discussion

The present study for the first time applied clinically relevant PCUS model in adult zebrafish, evaluating a wide range of behavioral and neurochemical alterations evoked by stress. The 11-week PCUS protocol developed here aimed to achieve more pronounced and stable behavioral and neurochemical alterations that may better fit the existing clinical data. Moreover, the present study also explored potential treatments of PCUS-evoked behavioral and neurochemical deficits, including both conventional (an SSRI fluoxetine) and novel putative (EPA) treatments, as well as the combination of PCUS with a pro-inflammatory agent LPS.

In general, our behavioral analyses revealed overt NTT anxiety induced by chronic stress exposure, as well as its efficient recovery by fluoxetine in the fluoxetine, fluoxetine + LPS and fluoxetine + EPA groups, but not by EPA or LPS. Fluoxetine alone increased top exploration vs. chronic stress or EPA and LPS exposure (but not fluoxetine + EPA and fluoxetine + LPS). Notably, other anxiety-related NTT endpoints were less sensitive to experimental manipulations.

Interestingly, PCUS stress alone did not alter locomotor endpoints, such as velocity and time spent mobile, whereas EPA, fluoxetine + EPA and fluoxetine + LPS showed hypolocomotion by reducing zebrafish velocity. While shorter CUS batteries affected zebrafish locomotion, these effects are conflicting, and include both hyper- and hypocolomotor^27^ phenotypes, likely representing psychomotor agitation or motor retardation, commonly reported in clinical affective disorders^31^. In contrast, elevated anxiety in zebrafish remains a strikingly consistent finding across multiple CUS studies^25–27^, including here, hence collectively reinforcing its high clinical and translational value as a hallmark affective phenotype evoked in zebrafish by chronic stress. Because anxiety is commonly seen in clinical patients exposed to chronic stress^32^, as well as in chronically stressed rodent models^33,34^, the later also emphasizes the value of elevated anxiety response as an evolutionarily conserved, ‘core’ affective phenotype across taxa that is nearly 400 million years old.

Likewise, PCUS reduced the ST average inter-fish distance in zebrafish, indicating increased anxiety and/or increased sociality^35,36^, whereas fluoxetine, EPA, and their combination all recovered this index. Although LPS alone did not worsen zebrafish group behavior (compared to PCUS fish), LPS + fluoxetine did (vs. any other group tested), suggesting some complex interplay between these compounds, likely involving inflammatory pathways.

Notably, PCUS somewhat increased time spent in light CPA area (indicating increased learning capabilities) compared to all other experimental groups, except the controls. As the CPA protocol is based on the avoidance stress response, alterations in baseline stress reactivity (e.g., baseline anxiety) may confound the phenotype observed here. For example, stressed fish may be more sensitive to stressful stimuli, and hence be more sensitive to conditional avoidance (and learn faster) than control fish. In line with this notion, all low-stress groups (fluoxetine, fluoxetine + EPA and fluoxetine + LPS) learned worse than the PCUS fish, as indicated by shorter time spent in the light compartment. However, other studies report poorer learning in zebrafish in a closely related inhibitory avoidance learning paradigm^37^, which suggests possible conceptual/construct differences between the tests. These effects may also correlate with increased density of dendritic spines observed in chronically stressed zebrafish that was recovered to the control levels following fluoxetine treatment^26^. Finally, CPA itself was successfully modeled here, as indicated by the light conditioning in control group vs. intact, experimentally naïve fish, thereby confirming the validity of the method used here.

Chronic fluoxetine treatment reversed most of PCUS behavioral effects and efficiently counteracted the effects of PCUS + LPS exposure, in line with anxiolytic^38^, antidepressant^39^ and anti-inflammatory^40^ effects observed for SSRIs clinically, hence supporting the overall translational validity of the PCUS model developed here. This finding also parallels fluoxetine effects in other zebrafish chronic stress studies, including the modulation of NTT anxiety and shoaling, observed after 5-week CUS^26,27^. Interestingly, while fluoxetine and LPS alone did not alter zebrafish locomotion, their combination produced hypolocomotion here. Furthermore, while fluoxetine normalized the ST inter-fish distance, and LPS did not affect this endpoint compared to stress group, their combination increased it, supporting complex interactions between the two compounds likely associated with altered inflammatory pathways in the brain.

Intriguingly, EPA alone did not recover zebrafish NTT anxiety-related behavior, whereas fluoxetine + EPA was close to both control and stressed groups, highlighting a weaker effect of the drug combination than that of fluoxetine alone. These results are at odds with some clinical studies on positive effects of EPA alone in depression^41,42^, and with the fact that fluoxetine + EPA is more efficient (vs. them alone) in such therapy^43^. The exact nature of these observed discrepancies in zebrafish is unclear. One possible explanation concerns overt metabolic differences between zebrafish and humans, and the fact that some EPA effects in human studies may be metabolic- and dietary-related^41,42^. However, EPA counteracted stress effects in ST, suggesting its correction of social deficits in stressed fish. Although fluoxetine efficiently counteracted PCUS + LPS-induced anxiety, given jointly with LPS, it produced hypolocomotion and shoal disruption not observed in either treatment group. Thus, further studies are needed to better understand the LPS effects in the PCUS zebrafish model developed here.

While disturbed serotonergic system is often observed in zebrafish models of stress^27,44^, serotonin was unaltered by PCUS here, likely because serotonergic changes may occur earlier then norepinephrinergic, given that all previous fish studies used shorter CUS models, whose neurochemical responses highly depend on the duration of stress^27^. Similarly, in rats, 6-week CUS decreases norepinephrine in PFC, hippocampus and hypothalamus, and increases dopamine levels in the same brain areas^45^, whereas 7-day CUS decreases dopamine and serotonin levels in PFC, striatum and hippocampus^46^. Norepinephrine levels rise in plasma of depressed patients^47,48^, and correlate with depressive and anxiety symptoms in healthy subjects^49^. At the same time, we recognize that rodent studies usually focus on various brain areas, whereas our study investigates whole-brain neurochemical changes, thus complicating direct cross-species comparisons.

In summary, our PCUS protocol induced anxiety-like effect that may be rescued with fluoxetine, but not EPA alone, and was not sufficiently worsened by exogenous pro-inflammatory modulation by LPS. While PCUS increased norepinephrine in zebrafish brain, fluoxetine or EPA restored its levels, fluoxetine decreased serotonin metabolism, EPA increased dopamine, and LPS increased both norepinephrine and dopamine levels. Finally, fluoxetine effectively recovered most of PCUS-evoked behavioral and neurochemical alterations alone and in combination with LPS or EPA.

In general, brain monoamines are major factors in chronic stress in fish, mammals^50^ and humans^51^. Here, PCUS significantly elevated only norepinephrine levels vs. controls. Given that stress increases the activity of the sympathetic nervous system, this observation parallels rodent data where stress also increases norepinephrine^52,53^. A widely used SSRI, fluoxetine, decreased 5-HIAA (the main metabolite of serotonin) in zebrafish brain, as well as the ratio of 5-HIAA/serotonin (vs. control group), reflecting lower serotonin turnover, a common biomarker of SSRI antidepressant action in zebrafish models^54,55^. In the present study, fluoxetine also normalized norepinephrine levels vs. PCUS alone and in combination with other drugs.

LPS, the main component of the membrane of gram-negative bacteria, can trigger inflammation via immune and non-immune mechanisms in vivo^56^, promoting the release of pro-inflammatory cytokines interleukin (IL) IL-1β and tumor necrosis factor-β (TNF-β)^57^. Proinflammatory cytokines are often associated with various mental illnesses, such as depression^58^. Although norepinephrine remained elevated (vs. control) by LPS, the latter also increased dopamine levels in the brain, suggesting the ability of LPS to promote zebrafish stress response, paralleling stress-potentiated dopamine activity in clinical and rodent models^59,60^.

LPS also modulates serotonin metabolism, for example, activating the serotonin transporter (SERT) and, consequently, serotonin reuptake^61,62^. Similar effect was observed here for LPS + fluoxetine, lowering serotonin levels (vs. chronic stress, fluoxetine, EPA and LPS), and also affecting serotonin turnover. Furthermore, the same combination also elevated dopamine turnover vs. LPS alone, which was interesting, given that both fluoxetine^63^ and LPS in our study increased brain dopamine levels. Finally, fluoxetine rescued norepinephrine levels even in combination with LPS, proving efficiency of this SSRI to counteract both inflammatory and stress-associated neurochemical alterations.

In general, EPA is a critical PUFA with multiple physiological functions in vivo. Unlike LPS, it exerts pronounced anti-inflammatory properties, beneficial in various psychiatric disorders^64,65^. The link of omega-3 PUFAs to dopamine and its metabolism has already been demonstrated^66^. In line with this, zebrafish treated with EPA in the present study had higher levels of dopamine and DOPAC/dopamine ratios here, and EPA effectively recovered norepinephrine levels. However, EPA evoked similar effects only in combination with fluoxetine, which not only normalized norepinephrine (elevated by PCUS), but also lowered 5-HIAA levels, and, like fluoxetine alone, decreased the 5-HIAA/serotonin ratio compared to control, thus promoting beneficial monoaminergic effects of fluoxetine. Collectively, our neurochemical results support the possibility that the combination of EPA + fluoxetine is more effective in the treatment of stress-related pathologies than these agents alone^67^.

However, there were also some conceptual and methodological limitations of the present study. For example, neurochemical analyses used here utilized whole-brain samples in contrast to the region-specific study of neurochemical changes and thus may mask neurochemical differences that are specific for brain areas. Furthermore, the zebrafish, like many other model organisms, exhibits pronounced intraspecies differences^68,69^, including sex differences^70^, that may influence stress and pharmacologically induced phenotypes. Although studying intraspecies variation was beyond the scope of the present study, this aspect of PUCS-evoked phenotypes clearly merits further scrutiny. Likewise, complementing our whole-brain HPLC analyses here, assessing more nuanced (brain region-specific) profiles of PCUS- and treatment-evoked neurochemical responses is also warranted.

Moreover, while most zebrafish CUS studies have shown anxiogenic phenotype^44,71,72^, as we did here as well, some reports failed to induce pronounced anxiety^73^, likely due to a short-lived nature of the affective syndrome they attempted to evoke. Given problems with data reliability and replicability in the field^74^, the choice of prolonged stress protocol, such as PUCS developed here, seems to be justified. Finally, while chronic stress models are widely interpreted as models of affective disorders, including depression, it is still unclear whether there is a clear-cut depression-like phenotype in the zebrafish^75^. Given the constantly evolving, and sometimes opposite, behavioral patterns evoked by chronic stress in many neurobehavioral domains (except anxiety)^26,27^, further studies are needed to better understand the exact interplay between these factors in CUS, necessitating further protocols with differing numbers of stressors, their severity, and modeling duration.

## Methods

### Animals and housing

Adult mature (5–7 months old) wild-type short-fin zebrafish of both sexes (approximately 50:50 ratio) were received from a local distributor (Tropic Aquarium, Ltd., St. Petersburg, Russia). Prior to testing, the fish were kept for at least 3 weeks under standard conditions in large 110-L plastic covered containers, with water temperature 27 ± 0.5 °C, pH 7.4, lighting (950–960 lux), adherence to day and night regimen, feeding twice a day with special feed pellets Neon Micro Granules for fish 1–2 cm in size (Dajana Pet, Bohuňovice, Czech Republic)^76^. Zebrafish were housed in the ZebTec Active Blue Stands with Water Treatment Unit (Tecniplast, West Chester, USA). All fish were from the same population, and were randomly divided into experimental groups using an online random number generator. The strain selection for the present study was based on population validity considerations and their relevance for the present study^77^. Specifically, while genetically controlled inbred zebrafish strains may offer reproducible and more reliable systems for neurogenetics research, modeling CNS disorders (such as in the present study) involves mimicking ‘real’ human maladies that affect genetically heterogenous clinical populations. Thus, using outbred populations of zebrafish can represent a more populationally valid and translationally relevant approach for the purpose of this study. This selection also considered overt strain-specific peculiarities of zebrafish behaviors in different tests^78^ that can be mitigated by using wild-type outbred fish, and also paralleled recent rodent findings (noting no higher phenotypic trait variability in outbred (vs. inbred) mice and concluding that outbred strains may be better subjects for most biomedical experiments^79^).

All animals tested were included in final analyses, without removing outliers. All experiments were performed as planned, and all analyses and endpoints assessed were included without omission. Animal experiments were approved by the Institutional IACUC and fully adhered to National and Institutional guidelines and regulations. The study experimental design and its description here, as well as data analysis and presenting, adhered to the ARRIVE (Animal Research: Reporting of In Vivo Experiments) guidelines for reporting animal research and the PREPARE (Planning Research and Experimental Procedures on Animals: Recommendations for Excellence) guidelines for planning animal research and testing.

### Prolonged chronic unpredictable stress (PCUS)

The experimental fish were kept in 4-L hometanks (20 fish/tank) and subjected to various stressors daily for the 11 weeks, similar to^26,27^, including crowding (10 fish/L) in 20-L buckets for 6 h, 1-min air exposure (by lifting the fish from their tanks by the net and leaving in the air), net chasing, cooling (to 10 °C), mild hypothermia (22 °C) and hyperthermia (35 °C) in 5-L jars, 3-cup crowding stress (12 fish/0.5-L cup), shoaling (placing 4–5 fish per NTT), predator (12-cm Oscar fish, *Astronotus ocellatus, *and 7-8-cm Blue marble gourami, *Trichogaster trichopterus*) or predator water exposure (water collected after 7-day housing from predator hometanks), exposure to a different (green GloFish) zebrafish strain, to novel objects (35 plastic 'Kinder surprise' toys), light–dark box (see above), shallow water (30–60% of the normal water level), 300-lux bright and 1500-lux extra-bright light (produced by two 60-wt light bulbs placed 20 or 2 cm above the water surface, respectively), electric shock (0.1 V/cm), noise (a 50-db drill sound from an online YouTube drilling video), shaking/vortexing for 30 s (10 fish/50-ml tube, at 1000 rmp), alarm pheromone exposure (0.5 mL/L), social isolation (1 fish/90-mL plastic cup), food deprivation and darkness (see Table 1 for details).

Control fish were housed similarly to the experimental cohort but remained experimentally naïve for the entire study duration. On Day 57, the stressed fish cohort was divided into six groups: the group continued to be exposed only to chronic stress, or groups exposed to chronic fluoxetine, eicosapentaenoic acid (EPA), lipopolysaccharide (LPS), fluoxetine + EPA), and fluoxetine + LPS for the final 3 weeks. Since LPS was injected intraperitoneally once a week, another group was added, where saline solution was injected intraperitoneally (injection control). As no behavioral alterations were observed between the groups, this additional control was excluded from further analyses (NS, data not shown).

Fluoxetine (Biocom Ltd., Stavropol, Russia) is a commonly used antidepressant clinically^80–82^ and has been tested extensively in various animal models, including rodents^83–86^ and zebrafish^25,26,87–89^. The duration of treatment, concentration and route of administration were selected based on the previous studies in stress-related models^26,50^. The test fish were kept in hometanks with 0.1 mg/L fluoxetine, and the water was changed daily, as in ^27^.

LPS from Escherichia coli O55:B5 (Sigma Aldrich, St. Louis, MO, USA), was chosen here for its ability to induce inflammation^90^. EPA was used here for its anti-inflammatory properties^91^. 10 μL LPS solution was injected intraperitoneally (once a week) and contained 3 μg LPS. During the injection procedure, the animals were briefly immobilized by a wet net on a wet sponge, and quickly injected through the net using a 26G needle without anesthesia, as described previously^92^. The dose were chosen based on zebrafish^93,94^ and rodent^95–99^ studies, and adjusted for chronic exposure. EPA (Tokyo Chemical Industry Co. Ltd., Tokyo, Japan) was fed to the fish with Neon Micro Granules (100 mg of EPA mixed with 7 g of standard fish fit used in the study, 10.5 g of gelatin, and 35 ml of water, as in ^100^). The EPA doses were adapted and extrapolated from rodent studies^101–105^. Mortalities observed due to the PCUS exposure and pharmacological manipulations are reported in Supplementary Table S3.

### Behavioral testing

Following an 11-week PCUS protocol, zebrafish behavioral and cognitive phenotypes were assessed in the novel tank test (NTT), shoaling test (ST), and the conditioned place aversion (CPA) test. Behavioral assays were organized in the order of increasing stress intensity, aiming to reduce the effect of the preceding testing^17^. Prior to testing, the fish were kept for 2 h in a testing room for acclimation, and were returned to the holding room after testing. Behavioral testing was performed between 11.00 and 17.00 h and was recorded with a SJ4000 action camera (SJCAM, Ltd., Shenzhen, China) at 60 frames/s. Experimenters were blinded to the treatments during behavioral testing and neurochemical analyses, including statistical and video analyses, and used individual codes for fish/groups identification. Manual analysis of behavioral data was performed by two highly-trained observers (blinded to the groups) with inter- and intra-rater reliability of > 0.85, as assessed by Spearman correlation as part of the laboratory’s standard operating procedure (SOP).

The NTT apparatus was an acrylic rectangular tank (20 height × 20 length × 5 width, cm), filled with water up to a 19-cm height, and divided into two equal virtual horizontal portions. The back and lateral sides of the tank were pasted with a white PVC envelope to increase contrast during behavioral recording. Each fish was recorded separately, immediately after being taken from the hometank, by a SJ4000 action camera for 5 min, assessinging velocity (cm/s), the number of top entries, time spent in top (s), and the latency to enter the top (s) of the tank^106^, using Noldus EthoVision XT11.5 software (Noldus IT, Wageningen, Netherlands).

The ST apparatus was similar to that of the NTT. During the testing, the fish from each group were placed in the tank in groups of 4–6, immediately after being taken from the hometank, and (after a 10-min acclimation to the apparatus) their shoals were photographed using a SJ4000 action camera every 60 s for 10 min. Each photo (total number: 198–541 photos per group) was next calibrated to the size of the tank, manually measuring the average inter-fish distance (cm)^107,108^.

The CPA apparatus was a plastic rectangular tank (12 long, 25 wide, 14 high cm) divided into two equal-sized black and light compartments filled with water. In the dark part, two metal plates were attached opposite to each other, connected to a custom-made current generator (0.1 V/cm). On Day 1 of the CPA test, the fish were trained by placing shoals of 10 fish in a CPA tank for 4 h with the generator turned on, supplying current to the dark section of the apparatus. On Day 2, each fish was recorded separately for 5 min with the current generator turned off, immediately after being taken from the hometank. In this assay, the time spent in the 'preferred' light zone (s) vs. 'punished' dark zone was assessed using Noldus EthoVision XT11.5 software.

### Neurochemical analyses

One day after the last behavioral experiment (shoaling test on Day 81 of PUCS protocol), the fish (n = 12 per group) were sacrificed in ice water followed by decapitation, their brains dissected on ice, frozen in liquid nitrogen, and stored at − 80 °C. Monoamines in adult zebrafish brain were assayed using the high-performance liquid chromatography (HPLC)^109,110^, and were chosen here as key stress biomarkers in various mental disorders in humans, as well as in rodent and zebrafish models^55,111^. The study examined norepinephrine, as well as serotonin, dopamine and their respective metabolites 5-HIAA and DOPAC.

All samples were weighed and placed in test tubes with ice-cold 10 μL of 0.1 M perchloric acid solution (Sigma Aldrich, St. Louis, MO, USA) per 1 mg of brain weight, with 100 ng/mL 3,4-dihydroxybenzylamine (DHBA, internal standard) to preserve neurochemical analytes^54^. For homogenization, the samples were sonicated for 10 s using a Qsonica sonicator (Qsonica, LLC, Newtown, CT, USA). The resulting homogenized product was centrifuged for 10 min (at 14,000 rpm in 4 °C), the supernatant was filtered through a Durapore-PVDF centrifuge filter with a pore size of 0.22 μm (Merck Millipore, Billerica, MA, USA)^54^. HPLC was performed on an HTEC-500 chromatograph (Eicom, San Diego, CA, USA) with a WE-3 G carbon electrode using an applied potential of + 650 mV with a CA-5ODS column. The mobile phase consisted of 0.1 M phosphate buffer, 400 mg/L sodium octyl sulfonate, 50 mg/L ethylenediamine tetra-acetic acid (EDTA), and 17% methanol. The required pH value (4.5) was adjusted with phosphoric acid. All reagents were purchased from Sigma Aldrich. Concentration data were normalized using individual DHBA concentrations in the samples, and are reported as pg/mg brain tissue weight. We also computed the 5-HIAA/serotonin and DOPAC/dopamine ratios (that reflect the metabolism/turnover of the corresponding monoamines in the brain), similar to^54^.

### Statistical analyses and data handling

Statistical analysis was performed using the Statistics 10 and GraphPad Prism 7 (GraphPad, San Diego, CA). Behavioral data were analyzed using the Kruskal–Wallis (KW) test, followed by Dunn's post-hoc test for pairwise group comparisons. Results are presented as median and interquartile range. The sample size was determined based on previous behavioral studies in zebrafish^26,54,55,109,112^. Initially, we started with n = 22 per group in all groups at the beginning of the PCUS protocol, although n’s decreased in some groups to 21 for control (due to injections) and PCUS, to 12–16 for the LPS, EPA and fluoxetine + LPS on the last day of the study (Supplementary Table 3S). All fish tested were included in the final analysis without attrition or exclusion, and all planned analyzes were presented here. All experimenters were unaware of the treatment groups during behavioral testing, neurochemical and genomic analyzes (including statistics and video analysis), using individual codes to identify fish/groups.

### Ethical confirmation statements

Animal experiments were approved by IACUC of St. Petersburg State University and fully adhered to the National and Institutional guidelines and regulations on animal experimentation, as well as the 3Rs principles of humane animal experimentation.

## Supplementary Information

Supplementary Information.

## Data Availability

The datasets generated and/or analyzed during the current study are available from the corresponding authors upon reasonable requests.
