# Supplementary material for: Modulation of behavioral and neurochemical responses of adult zebrafish by fluoxetine, eicosapentaenoic acid and lipopolysaccharide in the prolonged chronic unpredictable stress model
Source: Sci Rep. 2021 Jul 12;11:14289. doi: 10.1038/s41598-021-92422-6 (PMC8275758; doi:10.1038/s41598-021-92422-6)
Supplement: Supplementary file 1 — Supplementary Information. [file 41598_2021_92422_MOESM1_ESM.docx]

**Supplementary ONLINE materials**

**Paper:** **Modulation of behavioral and neurochemical responses in adult zebrafish by fluoxetine, eicosapentaenoic acid and lipopolysaccharide in the prolonged chronic unpredictable stress model**

Konstantin A. Demin*^+1,2^, Tatiana O. Kolesnikova^+1,7^, David S. Galstyan^+1,3^, Nataliya A. Krotova^1,2^, Nikita P. Ilyin^1^, Ksenia A. Derzhavina^1,2^, Nataliia A. Levchenko^1^, Tatyana Strekalova, Murilo S. de Abreu^6^, Elena V. Petersen^6^, Maria Seredinskaya^1^, Yulia V. Cherneyko^1^, Iurii M. Kositsyn^1^, Dmitry V. Sorokin^2^, Konstantin N. Zabegalov^1,7^, Mikael S. Mor^1^, Evgeniya V. Efimova^1^, and Allan V. Kalueff*^4,5,8^

^1^Institute of Translational Biomedicine, St. Petersburg State University, St. Petersburg, Russia

^2^Institute of Experimental Medicine, Almazov National Medical Research Centre, Ministry of Healthcare of Russian Federation, St. Petersburg, Russia

^3^Laboratory of Preclinical Bioscreening, Granov Russian Research Center of Radiology and Surgical Technologies, Ministry of Healthcare of Russian Federation, Pesochny, Russia

^4^School of Pharmacy, Southwest University, Chongqing, China;

^5^Ural Federal University, Ekaterinburg, Russia

^6^Moscow Institute of Physics and Technology, Moscow, Russia

^7^Neuroscience Program, Sirius University, Sochi, Russia

^8^University of Maastricht, Maasticht, Netherlands

^9^Granov Russian Research Center of Radiology and Surgical Technologies, Ministry of Healthcare of Russian Federation, Pesochny, Russia

**^+^These authors contributed equally to the study**

**^*^Co-corresponding authors:**

Allan V. Kalueff, Ph.D., School of Pharmacy, Southwest University, Chongqing, China. Tel/Fax: +1-240-899-9571 E-mail: [avkalueff@gmail.com](mailto:avkalueff@gmail.com)

Konstantin A. Demin, Institute of Translational Biomedicine SPBU, St. Petersburg State University, St. Petersburg, Russia. E-mail: [deminkasci@gmail.com](mailto:deminkasci@gmail.com)

**Data availability**

The datasets generated and/or analyzed in the present study are available from the original article, supplementary materials, or the corresponding author (upon reasonable requests, for use in collaborative research projects and/or for joint publications resulting from such projects).

**Supplementary Table S1.** Behavioral alterations induced by prolonged chronic unpredictable stress (PCUS) exposure and fluoxetine, EPA or LPS treatment in adult zebrafish tested in the novel tank test (velocity, time spent top, latency to enter top, number of top entries), shoaling test (interfish distance) and CPA (time spent in light compartment). Data is represented as post-hoc Dunn’s test p-values for significant Kruskal-Wallis data and median (IQR) (n=12-22, also see Fig. 2-3 and Table 2 for statistical details). C- control, S – PCUS, F – fluoxetine, E – eicosapentaenoic acid (EPA), L - lipopolysaccharide (LPS).

| **Velocity, cm/s** | | | | | | **df=6** | | **χ2=30.84** | | **P<0.0001** |
| --- | --- | --- | --- | --- | --- | --- | --- | --- | --- | --- |
|  | **C** | **S** | **F** | **E** | **L** | **F+E** | | **F+L** | |  |
| **Median (IQR)** | 4.4 (1.4) | 3.8 (2.4) | 3.8 (1.4) | 1.8 (2.3) | 3.25 (1.6) | 2.9 (2.08) | | 2.3 (2.8) | |  |
| **C** |  | 0.5330 | 0.8102 | **0.0007** | 0.1943 | **0.0010** | | **0.0002** | |  |
| **S** | 0.5330 |  | >0.999 | 0.8465 | >0.999 | >0.999 | | 0.3604 | |  |
| **F** | 0.8102 | >0.999 |  | 0.5155 | >0.999 | 0.9845 | | 0.2130 | |  |
| **E** | **0.0007** | 0.8465 | 0.5155 |  | >0.999 | >0.999 | | >0.999 | |  |
| **L** | 0.1943 | >0.999 | >0.999 | >0.999 |  | >0.999 | | >0.999 | |  |
| **F+E** | **0.0010** | >0.999 | 0.9845 | >0.999 | >0.999 |  | | >0.999 | |  |
| **F+L** | **0.0002** | 0.3604 | 0.2130 | >0.999 | >0.999 | >0.999 | |  | |  |
| **Time spent in top, s** | | | | | | **df=6** | | **χ2=41.81** | | **P<0.0001** |
|  | C | S | F | E | L | F+E | | F+L | |  |
| **Median (IQR)** | 130 (93) | 24.2 (102.3) | 102.4 (102.3) | 7.6 (18.7) | 6.5 (30.2) | 77.8 (108) | | 91.4 (192.5) | |  |
| **C** |  | **0.0003** | >0.999 | **0.0003** | **0.0003** | 0.1193 | | >0.999 | |  |
| **S** | **0.0003** |  | **0.0051** | >0.999 | >0.999 | >0.999 | | 0.3777 | |  |
| **F** | >0.999 | **0.0051** |  | **0.0044** | **0.0035** | 0.7595 | | >0.999 | |  |
| **E** | **0.0003** | >0.999 | **0.0044** |  | >0.999 | >0.999 | | 0.2600 | |  |
| **L** | **0.0003** | >0.999 | **0.0035** | >0.999 |  | 0.9581 | | 0.1638 | |  |
| **F+E** | 0.1193 | >0.999 | 0.7595 | >0.999 | 0.958601 |  | | >0.999 | |  |
| **F+L** | >0.999 | 0.3777 | >0.999 | 0.2600 | 0.1638 | >0.999 | |  | |  |
| **Latency to enter top, s** | | | | | | **df=6** | | **χ2=32.65** | | **P<0.0001** |
|  | **C** | **S** | **F** | **E** | **L** | **F+E** | | **F+L** | |  |
| **Median (IQR)** | 0 (0) | 0 (300) | 0.0 (0.00) | 0 (189.8) | 173.9 (300) | 0 (98.98) | | 0 (0) | |  |
| **C** |  | **0.0466** | >0.999 | >0.999 | 0.0836 | >0.999 | | >0.999 | |  |
| **S** | **0.0466** |  | **0.0057** | >0.999 | >0.999 | >0.999 | | 0.2647 | |  |
| **F** | >0.999 | **0.0057** |  | 0.4935 | **0.0157** | 0.6527 | | >0.999 | |  |
| **E** | >0.999 | >0.999 | 0.4935 |  | >0.999 | >0.999 | | >0.999 | |  |
| **L** | 0.0836 | >0.999 | **0.0157** | >0.999 |  | >0.999 | | 0.3005 | |  |
| **F+E** | >0.999 | >0.999 | 0.6527 | >0.999 | >0.999 |  | | >0.999 | |  |
| **F+L** | >0.999 | 0.2647 | >0.999 | >0.999 | 0.3005 | >0.999 | |  | |  |
| **Number of top entries** | | | | | | | **df=6** | | **χ2=22.10** | **P<0.005** |
|  | **C** | **S** | **F** | **E** | **L** | **F+E** | | **F+L** | |  |
| **Median (IQR)** | 6.5 (9.25) | 3 (7) | 6.5 (6) | 1.5 (2.5) | 1.5 (5.75) | 4 (8) | | 2 (3) | |  |
| **C** |  | 0.0906 | >0.999 | 0.0758 | 0.1384 | >0.999 | | **0.0395** | |  |
| **S** | 0.0906 |  | 0.2404 | >0.999 | >0.999 | >0.999 | | >0.999 | |  |
| **F** | >0.999 | 0.2404 |  | 0.1912 | 0.3082 | >0.999 | | 0.0996 | |  |
| **E** | 0.0758 | >0.999 | 0.1912 |  | >0.999 | >0.999 | | >0.999 | |  |
| **L** | 0.1384 | >0.999 | 0.3082 | >0.999 |  | >0.999 | | >0.999 | |  |
| **F+E** | >0.999 | >0.999 | >0.999 | >0.999 | >0.999 |  | | >0.999 | |  |
| **F+L** | **0.0395** | >0.999 | 0.0996 | >0.999 | >0.999 | >0.999 | |  | |  |
| **Inter-fish distance, cm** | | | | | | **df=6** | | **χ2=162.25** | | **P<0.0001** |
|  | **C** | **S** | **F** | **E** | **L** | **F+E** | | **F+L** | |  |
| **Median (IQR)** | 4.1(3.7) | 2.6(2.9) | 4.5(5.52) | 3.52(3.5) | 2.9(3.48) | 3.6(5.1) | | 6.4(8.8) | |  |
| **C** |  | **<0.0001** | >0.999 | 0.142081 | **<0.0001** | >0.999 | | **0.0014** | |  |
| **S** | **<0.0001** |  | **<0.0001** | **0.0051** | >0.999 | **<0.0001** | | **<0.0001** | |  |
| **F** | >0.999 | **<0.0001** |  | **0.0007** | **<0.0001** | 0.0781 | | **0.0420** | |  |
| **E** | 0.1420 | **0.0051** | **0.0007** |  | >0.999 | >0.999 | | **<0.0001** | |  |
| **L** | **<0.0001** | >0.999 | **<0.0001** | >0.999 |  | **0.0042** | | **<0.0001** | |  |
| **F+E** | >0.999 | **<0.0001** | 0.0781 | >0.999 | **0.0042** |  | | **<0.0001** | |  |
| **F+L** | **0.0014** | **<0.0001** | **0.0420** | **<0.0001** | **<0.0001** | **<0.0001** | |  | |  |
| **Time spent in light compartment, s** | | | | | | **df=7** | | **χ2=36.86** | | **P<0.0001** |
|  | **C** | **I** | **S** | **F** | **E** | **L** | | **F+E** | | **F+L** |
| **Median (IQR)** | 191(139) | 72.7(33.2) | 245.3(122.9) | 137.5(75.6) | 152.3(152.5) | 141.9(109) | | 93.1(58.4) | | 112(87.9) |
| **C** |  | **0.0026** | >0.999 | >0.999 | >0.999 | >0.999 | | 0.1352 | | >0.999 |
| **I** | **0.0026** |  | **<0.0001** | 0.4438 | >0.999 | >0.999 | | >0.999 | | >0.999 |
| **S** | >0.999 | **<0.0001** |  | **0.0489** | 0.1510 | 0.2078 | | **0.0006** | | **0.0462** |
| **F** | >0.999 | 0.4438 | **0.0489** |  | >0.999 | >0.999 | | >0.999 | | >0.999 |
| **E** | >0.999 | >0.999 | 0.1510 | >0.999 |  | >0.999 | | >0.999 | | >0.999 |
| **L** | >0.999 | >0.999 | 0.2078 | >0.999 | >0.999 |  | | >0.999 | | >0.999 |
| **F+E** | 0.1352 | >0.999 | **0.0006** | >0.999 | >0.999 | >0.999 | |  | | >0.999 |
| **F+L** | >0.999 | >0.999 | **0.0462** | >0.999 | >0.999 | >0.999 | | >0.999 | |  |

**Supplementary Table S2.** Neurochemical alterations induced by prolonged chronic unpredictable stress (PCUS) exposure and fluoxetine, EPA or LPS treatment in adult zebrafish brain analyzed using high-performance liquid chromatography (HPLC). Data is represented as post-hoc Dunn’s test p-values for significant Kruskal-Wallis data and median (IQR) (n=12-22, also see Fig. 2-3 and Table 2 for statistical details). C- control, S – PCUS, F – fluoxetine, E – eicosapentaenoic acid (EPA), L - lipopolysaccharide (LPS), DOPAC - 3,4-dihydroxyphenylacetic acid, 5-HIAA - 5-hydroxyindoleacetic acid, HVA - homovanillic acid

| **Norepinephrine, pg/mg** | | | | | **df=6** | **χ2=20.45** | **P<0.005** |
| --- | --- | --- | --- | --- | --- | --- | --- |
|  | **C** | **S** | **F** | **E** | **L** | **F+E** | **F+L** |
| **Median (IQR)** | 1243 (356) | 1949 (723) | 1778 (1186) | 1580 (715) | 2109 (561) | 1456 (635) | 1278 (1067) |
| **C** |  | **0.0357** | 0.3927 | 0.7495 | **0.0036** | >0.999 | >0.999 |
| **S** | **0.0357** |  | >0.999 | >0.999 | >0.999 | >0.999 | >0.999 |
| **F** | 0.3927 | >0.999 |  | >0.999 | >0.999 | >0.999 | >0.999 |
| **E** | 0.7495 | >0.999 | >0.999 |  | >0.999 | >0.999 | >0.999 |
| **L** | **0.0036** | >0.999 | >0.999 | >0.999 |  | 0.8135 | 0.0515 |
| **F+E** | >0.999 | >0.999 | >0.999 | >0.999 | 0.8135 |  | >0.999 |
| **F+L** | >0.999 | >0.999 | >0.999 | >0.999 | 0.0515 | >0.999 |  |
| **Dopamine, pg/mg** | | | | | **df=6** | **χ2=18.7** | **P<0.005** |
|  | C | S | F | E | L | F+E | F+L |
| **Median (IQR)** | 153 (173.8) | 326 (198.6) | 311.9 (199.2) | 391 (189.2) | 379.8 (346.4) | 269.4 (163.1) | 191.1 (204.9) |
| **C** |  | 0.6616 | 0.9941 | **0.0400** | **0.0252** | >0.999 | >0.999 |
| **S** | 0.6616 |  | >0.999 | >0.999 | >0.999 | >0.999 | >0.999 |
| **F** | 0.9941 | >0.999 |  | >0.999 | >0.999 | >0.999 | >0.999 |
| **E** | **0.0400** | >0.999 | >0.999 |  | >0.999 | 0.9746 | 0.1806 |
| **L** | **0.0252** | >0.999 | >0.999 | >0.999 |  | 0.7044 | 0.1208 |
| **F+E** | >0.999 | >0.999 | >0.999 | 0.9746 | 0.7044 |  | >0.999 |
| **F+L** | >0.999 | >0.999 | >0.999 | 0.1806 | 0.1208 | >0.999 |  |
| **DOPAC, pg/mg** | | | | | **df=6** | **χ2=7.499** | **P=0.2772** |
|  | **C** | **S** | **F** | **E** | **L** | **F+E** | **F+L** |
| **Median (IQR)** | 47.48 (24.12) | 58.62 (14.61) | 47.65 (34.57) | 43.86 (21.98) | 46.8 (21.98) | 45 (21.44) | 69.17 (46.72) |
| **C** |  | >0.999 | >0.999 | >0.999 | >0.999 | >0.999 | >0.999 |
| **S** | >0.999 |  | >0.999 | >0.999 | >0.999 | >0.999 | >0.999 |
| **F** | >0.999 | >0.999 |  | >0.999 | >0.999 | >0.999 | >0.999 |
| **E** | >0.999 | >0.999 | >0.999 |  | >0.999 | >0.999 | >0.999 |
| **L** | >0.999 | >0.999 | >0.999 | >0.999 |  | >0.999 | >0.999 |
| **F+E** | >0.999 | >0.999 | >0.999 | >0.999 | >0.999 |  | >0.999 |
| **F+L** | >0.999 | >0.999 | >0.999 | >0.999 | >0.999 | >0.999 |  |
| **Serotonin, pg/mg** | | | | | **df=6** | **χ2=34.71** | **P<0.0001** |
|  | **C** | **S** | **F** | **E** | **L** | **F+E** | **F+L** |
| **Median (IQR)** | 108.3 (173.9) | 223.9 (140.2) | 201.8 (121.4) | 201.1 (153.9) | 277.8 (91.3) | 149.7 (56.9) | 67.73 (37.77) |
| **C** |  | >0.999 | >0.999 | >0.999 | 0.1090 | >0.999 | 0.5952 |
| **S** | >0.999 |  | >0.999 | >0.999 | >0.999 | 0.8302 | **0.0011** |
| **F** | >0.999 | >0.999 |  | >0.999 | >0.999 | >0.999 | **0.0020** |
| **E** | >0.999 | >0.999 | >0.999 |  | >0.999 | >0.999 | **0.0020** |
| **L** | 0.1090 | >0.999 | >0.999 | >0.999 |  | >0.999 | **<0.0001** |
| **F+E** | >0.999 | 0.8302 | >0.999 | >0.999 | >0.999 |  | 0.9746 |
| **F+L** | 0.5952 | **0.0011** | **0.0020** | **0.0020** | **<0.0001** | 0.9746 |  |
| **5HIAA, pg/mg** | | | | | **df=6** | **χ2=33.11** | **P<0.0001** |
|  | **C** | **S** | **F** | **E** | **L** | **F+E** | **F+L** |
| **Median (IQR)** | 143.2 (70.9) | 160.3 (44.7) | 97.33 (71.06) | 129.8 (73.53) | 150.1 (32.6) | 70.02 (28.29) | 109.6 (88.22) |
| **C** |  | >0.999 | 0.4589 | >0.999 | >0.999 | **0.0039** | >0.999 |
| **S** | >0.999 |  | **0.0337** | >0.999 | >0.999 | **<0.0001** | 0.8135 |
| **F** | 0.4589 | **0.0337** |  | >0.999 | >0.999 | >0.999 | >0.999 |
| **E** | >0.999 | >0.999 | >0.999 |  | >0.999 | 0.1518 | >0.999 |
| **L** | >0.999 | >0.999 | >0.999 | >0.999 |  | **0.0002** | >0.999 |
| **F+E** | **0.0039** | **<0.0001** | >0.999 | 0.1518 | **0.0002** |  | 0.2357 |
| **F+L** | >0.999 | 0.8135 | >0.999 | >0.999 | >0.999 | 0.2357 |  |
| **5HIAA to serotonin ratio** | | | | | **df=6** | **χ2=33.31** | **P<0.0001** |
|  | **C** | **S** | **F** | **E** | **L** | **F+E** | **F+L** |
| **Median (IQR)** | 1.143 (0.8) | 0.68 (0.39) | 0.41 (0.2) | 0.58 (0.31) | 0.56 (0.28) | 0.41 (0.47) | 1.63 (0.63) |
| **C** |  | >0.999 | **0.0166** | 0.5010 | >0.999 | 0.1556 | >0.999 |
| **S** | >0.999 |  | 0.5582 | >0.999 | >0.999 | >0.999 | 0.1943 |
| **F** | **0.0166** | 0.5582 |  | >0.999 | >0.999 | >0.999 | **<0.0001** |
| **E** | 0.5010 | >0.999 | >0.999 |  | >0.999 | >0.999 | **0.0041** |
| **L** | >0.999 | >0.999 | >0.999 | >0.999 |  | >0.999 | **<0.0001** |
| **F+E** | 0.1556 | >0.999 | >0.999 | >0.999 | >0.999 |  | **0.0041** |
| **F+L** | >0.999 | 0.1943 | **<0.0001** | **0.0041** | **0.0041** | **0.0041** |  |
| **DOPAC to dopamine ratio** | | | | | **df=6** | **χ2=24.76** | **P=0.0004** |
|  | **C** | **S** | **F** | **E** | **L** | **F+E** | **F+L** |
| **Median (IQR)** | 0.26 (0.19) | 0.16 (0.08) | 0.18 (0.07) | 0.12 (0.07) | 0.13 (0.08) | 0.19 (0.22) | 0.31 (0.23) |
| **C** |  | >0.999 | >0.999 | >0.999 | 0.0659 | >0.999 | >0.999 |
| **S** | >0.999 |  | >0.999 | >0.999 | >0.999 | >0.999 | 0.2848 |
| **F** | >0.999 | >0.999 |  | >0.999 | >0.999 | >0.999 | >0.999 |
| **E** | >0.999 | >0.999 | >0.999 |  | >0.999 | >0.999 | **0.0021** |
| **L** | 0.0659 | >0.999 | >0.999 | >0.999 |  | >0.999 | **0.0021** |
| **F+E** | >0.999 | >0.999 | >0.999 | >0.999 | >0.999 |  | 0.4489 |
| **F+L** | >0.999 | 0.2848 | >0.999 | **0.0021** | **0.0021** | 0.4489 |  |

**Supplementary Table S3.** Mortalities observed due to the prolonged chronic unpredictable stress (PCUS) exposure and fluoxetine, EPA or LPS treatments in adult zebrafish used in the present study (original n’s at the beginning if the study = 22).

| **Groups** | **Mortality (fish) per group** |
| --- | --- |
| Control | 0 |
| PCUS | 1 |
| Fluoxetine | 0 |
| Eicosapentaenoic acid | 6 |
| Lipopolysaccharide | 10 |
| Fluoxetine+Eicosapentaenoic acid | 0 |
| Fluoxetine+ Lipopolysaccharide | 9 |

**CRediT authorship contribution statement**

Konstantin A. Demin (Investigation) (Methodology) (Project administration) (Validation) (Visualization) (Writing - original draft) (Writing - review and editing), Tatiana O. Kolesnikova (Visualization) (Investigation) (Methodology) (Formal analysis) (Writing - Original Draft) (Writing - review and editing), David S. Galstyan (Investigation) (Methodology) (Formal analysis) (Writing - review and editing) (Writing - Original Draft), Nataliya A. Krotova (Investigation) (Methodology) (Writing - review and editing), Nikita P. Ilyin (Investigation) (Writing - review and editing), Ksenia A. Derzhavina (Investigation) (Writing - review and editing), Nataliia A. Levchenko (Investigation) (Writing - review and editing), Tatyana Strekalova (Investigation) (Writing - review and editing), Murilo S. de Abreu (Investigation) (Writing - review and editing), Elena V. Petersen (Investigation) (Writing - review and editing), Maria Seredinskaya (Investigation) (Writing - review and editing), Yulia V. Cherneyko (Investigation) (Writing - review and editing, Yuriy M. Kositsyn (Investigation) (Writing - review and editing), Dmitry V. Sorokin (Investigation) (Writing - review and editing), Konstantin N. Zabegalov (Investigation) (Writing - review and editing), Mikael S. Mor (Methodology) (Writing - review and editing), Evgeniya V. Efimova (Methodology) (Writing - review and editing), and Allan V. Kalueff (Conceptualization) (Funding acquisition) (Methodology) (Project administration) (Resources) (Supervision) (Validation) (Writing - review and editing).
